# Cerebrovascular Strokes During Venoarterial Extracorporeal Membrane Oxygenation

**DOI:** 10.1155/ccrp/9058296

**Published:** 2025-10-14

**Authors:** Zohair Al-Halees, Mosleh Nazzal Alanazi, Patricia Machado, Mary Jane Maghirang, Emad Hakami, Farouk Mostafa Faris, Michelle Gretchen Lo, Mohamed Laimoud

**Affiliations:** ^1^Cardiac Surgery Department, King Faisal Specialist Hospital and Research Center, Riyadh, Saudi Arabia; ^2^Cardiovascular Critical Care Department, King Faisal Specialist Hospital and Research Center, Riyadh, Saudi Arabia; ^3^Cardiovascular Nursing Department, King Faisal Specialist Hospital and Research Center, Riyadh, Saudi Arabia; ^4^Critical Care Medicine Department, Cairo University, Cairo, Egypt; ^5^Cardiovascular Critical Care Department, Prince Sultan Cardiac Center, Riyadh, Saudi Arabia

**Keywords:** cerebrovascular strokes, extracorporeal membrane oxygenation, intracranial hemorrhage (ICH), ischemic stroke, lactate, lactate clearance, mortality

## Abstract

**Background:**

Venoarterial extracorporeal membrane oxygenation (VA-ECMO) is a life-saving mechanical support in patients with cardiogenic shock. There are great variations in the reported rates of neurological complications and associated mortality. Our aim was to analyze our cohort of adult patients supported with VA-ECMO to identify the incidence, outcomes, and predictors of acute ischemic and hemorrhagic strokes.

**Methods:**

A total of 195 patients between January 2016 and January 2023 were reviewed, 22 (11.3%) ECPR patients were excluded, and 173 (88.7%) patients were analyzed. We divided the patients into stroke and nonstroke groups according to the presence of radiologically confirmed acute ischemic and hemorrhagic strokes.

**Results:**

Thirty-five (20.2%) patients had acute cerebrovascular strokes. 13 (7.5%) patients had intracranial hemorrhage (ICH) while 22 (12.7%) patients had ischemic stroke. The median age was 48 years (IQR: 31, 56), 98 (56.6%) patients were males, and 152 (87.9%) patients had cardiac surgeries. The patients who developed cerebrovascular stroke had higher blood lactate at ECMO initiation (8.9 [5.5, 11.2] versus 5.7 [4.6, 11.9] mmol/L, *p* = 0.02) and 12 h later (8.7 [4.7, 14.5] versus 5.8 [4.6, 15] mmol/L, *p* = 0.024) with lesser lactate clearance (LC) at 12 h (6.35 [−51.5, 40.6] versus 14.65% [−43.55, 38.3], *p* < 001) compared to the patients in the nonstroke group. The stroke group had longer ICU stay (21 vs. 15.5 days, *p* = 0.03), higher frequency of new hemodialysis (62.9% vs. 46.4%, *p* = 0.026), and on-ECMO mortality (54.3% vs. 44.9%, *p* = 0.041) compared with the nonstroke group. The ICH was associated with higher hospital mortality (*p* = 0.021) compared to the ischemic stroke. Logistic multivariate regression revealed that the initial lactate level (OR: 1.6, 95% CI: 1.2–8.92, *p* = 0.031), cardiopulmonary bypass time (OR:1.8, 95% CI: 1.32–6.42, *p* = 0.02), and LC at 12 h (OR: 2.4, 95% CI: 1.91–17.4, *p* = 0.042) were associated with ischemic stroke. Thrombocytopenia (OR: 3.22, 95% CI: 1.82–7.83, *p* = 0.001) and low body mass index (OR: 2.1, 95% CI: 1.31–4.6, *p* = 0.02) were associated with ICH.

**Conclusions:**

Ischemic and hemorrhagic strokes are frequent with VA-ECMO support and associated with worse outcomes, especially the hemorrhagic type. Awareness of the incidence and the factors associated with strokes is crucial in early identification and management.

## 1. Background

Venoarterial extracorporeal membrane oxygenation (VA-ECMO) is a rescue mechanical circulatory support effective in patients with refractory cardiogenic shock [1, 2]. The neurological complications that occur during VA-ECMO support include hypoxic–ischemic brain injury (HIBI), ischemic stroke, intracranial hemorrhage (ICH), and brain death [3]. The underlying pathophysiological mechanisms include reduced cerebral blood flow, thromboembolism from the heart or ECMO circuit, differential hypoxemia (Harlequin syndrome), and coagulation disarrangement [4–6].

Hwang et al. analyzed 20,297 adult patients who received VA-ECMO support at 534 centers between 2012 and 2021 from the Extracorporeal Life Support Organization (ELSO) registry and reported the increased incidence of ischemic stroke with associated increased 90-day mortality (65% vs. 36% mortality of those without ischemic stroke, *p* < 0.0001) [7]. There was a stable incidence of hemorrhagic stroke but with a significant 90-day mortality (72% vs. 37% for patients without ICH, *p* < 0.0001) [7].

There are great variations in the reported rates of neurological complications with VA-ECMO (7.4%–19%) and associated mortality (57%–85%) due to different protocols of neurological monitoring with study designs, including patients with extracorporeal cardiopulmonary resuscitation (ECPR) or inclusion of patients with VA-ECMO only or VA-and VV-ECMO [8–14]. However, the true incidence of neurological complications is much higher than the reported data. Postmortem studies revealed that 68%–90% of VA-ECMO nonsurvivors had cerebral infarcts which criticizes the neurological monitoring and detection of brain insults during VA-ECMO management [15–17].

Current gaps for neurological care during VA-ECMO include optimal mean blood pressure and anticoagulation to avoid/manage strokes, degree of safe changes of oxygen, carbon dioxide, acid–base status, body temperature to avoid brain insults, sedation protocols and interruptions, efficacy and frequency of neurological assessment, anticoagulation management to decrease thromboembolic and hemorrhagic risks, and limitations of brain imaging scans [18]. We conducted an observational retrospective analysis of the adult patients who received VA-ECMO in our center to identify the incidence, outcomes, and predictors of acute ischemic and hemorrhagic strokes.

## 2. Methods

### 2.1. Study Design and Population

The study was a single center cohort analysis of all patients ≥ 18 years who had VA-ECMO support between 2016 and 2023. Cerebral infarction and ICH were diagnosed by brain computed tomography (CT). All patients who had cardiac arrest and VA-ECMO initiation as ECPR were excluded from the study.

### 2.2. ECMO Implementation and Neurological Monitoring

All patients with VA-ECMO support had close neurological monitoring by the bedside nurse and the attending intensivist. Checking pupils' size and reactivity was routinely done every 2 h. Daily sedation withdrawal was being done for assessment of motor power, response to verbal and painful stimuli, and presence of motor deficit or asymmetry. Radiological confirmation was done by cerebral CT in case of abnormal neurological findings.

All patients with VA-ECMO support underwent monitoring of cerebral oxygenation (rSO2%) via forehead probes and near-infrared spectroscopy (NIRS) technique. Cerebral NIRS helps as an alert monitor to early detect occurrence of acute cerebral injury by drop of rSO2% > 25% of baseline or left–right rSO2 difference > 10% [19, 20]. Electroencephalography (EEG) was used for patients with clinical or subclinical seizures to help initiation and adjustment of antiepileptic drugs. As per our center policy, we use anticoagulation with unfractionated heparin infusion except in rare cases with heparin-induced thrombocytopenia, and we use argatroban infusion.

### 2.3. The Studied Variables

The baseline clinical variables included age, sex, underlying cardiac and cerebrovascular diseases, body mass index (BMI), cardiac surgery details, and cannulation approaches of ECMO. The laboratory variables included arterial blood lactate, platelet count, hemoglobin level, serum creatinine, liver enzymes, and bilirubin. The Sequential Organ Failure Assessment (SOFA) score was calculated for the patients studied at ECMO initiation then 48 h and 96 h later. The SOFA score consists of 6 variables representing different organs (Glasgow Coma Scale, oxygenation (PaO2/FiO2), mean arterial blood pressure (MAP), serum creatinine and bilirubin, and platelet count). The SOFA score reflects clinical severity and organs' affection. So, it was used in critically ill patients including those who required VA-ECMO support [21, 22]. The survival after VA-ECMO (SAVE) score was calculated before ECMO initiation for the enrolled patients. The SAVE score consists of many variables including the etiology of shock, hemodynamic profile, patient age and weight, use of mechanical ventilation before ECMO, and peak inspiratory pressure [23].

Lactate clearance (LC) was calculated at 12 h and 24 h after ECMO initiation [24, 25].

(1) $$\begin{array}{c} \text{LC at }12\text{ or }24\;h=\left[\left(\text{Lactate at }12\text{ or }24\;h-\text{initial lactate}\right)÷\text{initial lactate}\right]\times 100. \end{array}$$

Based on the CT findings, the studied patients were divided into stroke and nonstroke groups. Further subgrouping of the stroke group was done into the ischemic and hemorrhagic groups. The primary outcome was hospital mortality, and the secondary outcomes were the need for renal replacement therapy and length of stay.

### 2.4. Statistical Analysis

Data were checked for normality and presented as medians with interquartile range (IQR) due to skewed distribution. Categorical variables were described as frequencies with percentages and compared using the Chi-square test. Comparisons of continuous variables were done using the Mann–Whitney *U*-test. Multivariable regression models were done to get independent predictors of ischemic and hemorrhagic strokes. The Statistical Package for Social Sciences (SPSS) Version 28 (IBM Corp., USA) was used, and significance was determined as two-sided*p* < 0.05.

## 3. Results

### 3.1. Demographic and Clinical Data

**Figure 1:**
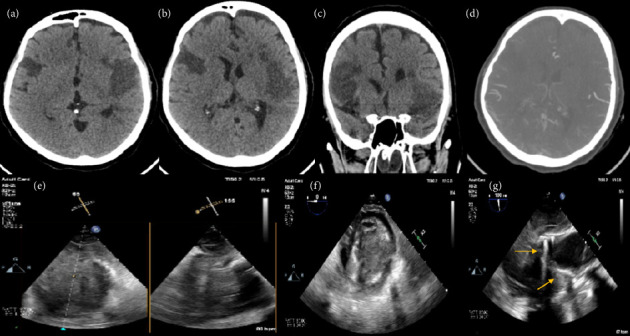
CT brain (a–d) and TEE (e–g) images of a 55-year-old woman developed postcardiotomy cardiogenic shock and required central VA-ECMO implantation. Despite left ventricular venting and anticoagulation, she developed thrombosis of the left ventricle and aortic root (e and f) with a stuck aortic valve (g, yellow arrows). NIRS continuous monitoring showed an abrupt decrease > 30%, emergency CT brain revealed bilateral ischemic infarcts (a–c), and angiography revealed patent middle cerebral arteries with paucity of the distal branches at areas of strokes (d).

**Figure 2:**
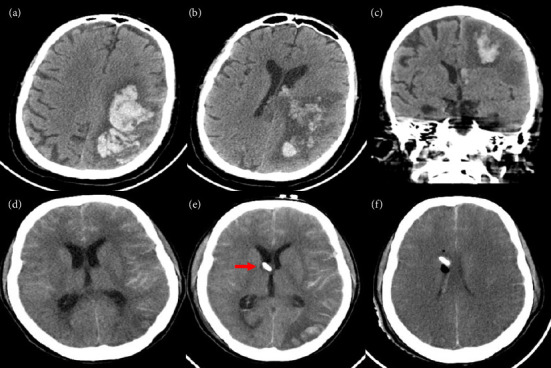
CT brain images (a–c) of a 56-year-old man presented with electrical storm that required VA-ECMO support and developed ICH after 6 days of ECMO support. CT brain images (d–f) of a 28-year-old female who developed subarachnoid hemorrhage (SAH) after 4 days of VA-ECMO support for cardiogenic shock due to severe pulmonary hypertension. Brain death occurred after SAH despite ventricular drain (red arrow).

Thirty-five (20.2%) patients had acute cerebrovascular stroke. 13 (7.5%) patients had ICH while 22 (12.7%) patients had ischemic stroke. The median age was 48 years (IQR: 31, 56), 98 (56.6%) patients were males, and 152 (87.9%) patients had cardiac surgeries. The preoperative demographic, clinical variables, cannulation approaches, and scores are summarized in Table 1 without statistically significant differences between the patients who developed stroke and those who did not (Table 1, and Figures 1 and 2).

### 3.2. Laboratory Data of the Patients Studied

**Figure 3:**
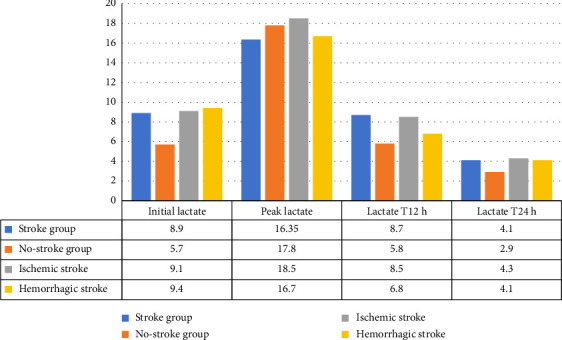
Median blood lactate levels (mmol/L) at VA-ECMO initiation, peak, and at 12 h and 24 h after ECMO.

**Figure 4:**
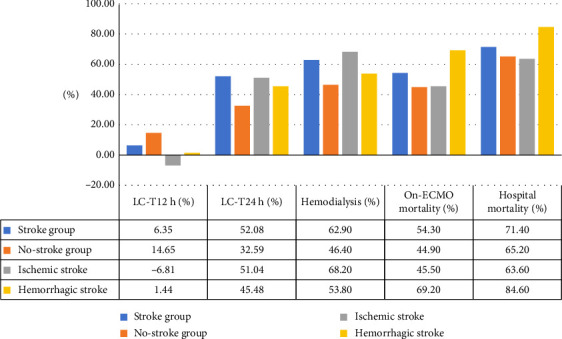
Percent of lactate clearance, mortality, and new need for hemodialysis.

The patients who developed acute cerebrovascular stroke had higher blood lactate at ECMO initiation (8.9 (5.5, 11.2) versus 5.7 [4.6, 11.9] mmol/L, *p* = 0.02) and 12 h later (8.7 [4.7, 14.5] versus 5.8 [4.6, 15] mmol/L, *p* = 0.024) with lesser LC at 12 h (6.35 [−51.5, 40.6] versus 14.65% [−43.55, 38.3], *p* < 001) compared to the patients in the nonstroke group. They had increased LC at the 24 h point (52.08 [5.88, 64.43] versus 32.59% [−17.76, 65.52], *p* = 0.003) indicating a delayed LC after ECMO support. There were insignificant differences between the two groups regarding the other laboratory variables and follow-up SOFA scores (Table 2, Figures 3 and 4).

Subgroup analysis showed that the patients who developed ICH were younger (29 [25, 51] versus 45 [31, 56] years, *p* = 0.04) with a lower BMI (22.7 [19.7, 29.2] versus 25.9 ]22.6, 31.6] kg/m^2^, *p* = 0.02) and had significant thrombocytopenia (32 [28, 67] versus 78 [54, 121.6] 10^9^/L, *p* = 0.001) and hypofibrinogenemia (1.21 [0.8, 1.92] versus 2.34 [1.74, 2.46] g/L, *p* = 0.04) 48 h after ECMO support compared to the patients without ICH, respectively. The patients who developed acute ischemic stroke had a prolonged CPB (228.5 [169, 319] versus 179 [148, 241] min, *p* = 0.042), higher blood lactate levels at ECMO initiation (9.1 [5.7, 12] versus 7.45 [5.1, 9.9] mmol/L, *p* = 0.023), at 12 h (8.5 [4.8, 14.8] versus 5.3 [4.3, 15] mmol/L, *p* = 0.04), with lesser LC at 12 h (−6.81 [−65.29, 41.94] versus 11.24% [−42.05, 38.3], *p* < 0.001) compared to the patients without ischemic stroke, respectively (Table 3).

### 3.3. Clinical Outcomes

The patients who developed stroke had significantly prolonged ICU stay (21 [12,38] versus 15.5 [8,26] days, *p* = 0.03), higher rate of new CRRT (62.9 versus 46.4%, *p* = 0.026), and higher on-ECMO mortality (54.3% versus 44.9%, *p* = 0.041) in comparison to the nonstroke group, respectively (Table 4).

The patients who developed ischemic stroke had significantly prolonged ICU stay (22.5 [13.5, 38] versus 16 [8, 31.5] days, *p* = 0.04) and higher rate of CRRT use (68.2 versus 47%, *p* = 0.004) compared to the remaining study population. Patients who developed ICH had significantly higher on-ECMO mortality (69.2 vs.45%, *p* = 0.03) and in-hospital mortality (84.6 versus 65%, *p* = 0.021) compared to the remaining study population. Patients with ICH had significantly greater mortality compared to the patients with ischemic strokes (Tables 5 and 6).

### 3.4. Predictors of Acute Cerebrovascular Strokes

Initial blood lactate (OR: 1.6, *p* = 0.031, 95% CI: 1.2–8.92), CPB time (OR: 1.8, *p* = 0.02, 95% CI: 1.32–6.42) and LC at 12 h (OR: 2.4, *p* = 0.042, 95% CI: 1.91–17.4), were the factors associated with the ischemic stroke. Thrombocytopenia (OR: 3.22, *p* = 0.001, 95% CI: 1.82–7.83) and low BMI (OR: 2.1, *p* = 0.02, 95% CI: 1.31–4.6) were the factors associated with ICH. The increase of blood fibrinogen level was protective against ICH but without a statistical significance (Table 7).

## 4. Discussion

While VA-ECMO is a critical intervention for treating cardiogenic shock, it poses significant risks of multiple organ damage, which can adversely impact patient outcomes. The reported rates of neurological complications and the associated mortality rates vary widely. This study aimed to evaluate our cohort of VA-ECMO patients to determine the incidence, outcomes, and factors associated with acute neurological strokes.

The actual incidence of neurological complications might be underestimated. Many patients could have died with multiorgan system failure without undergoing neuroimaging. Additionally, in other cases, the absence of regular standardized neurological monitoring may have concealed the true occurrence of these complications [3]. In our study, out of 173 adult patients mostly after cardiac surgery, 35 (20.2%) patients developed acute cerebrovascular stroke during VA-ECMO support. 22 (12.7%) patients developed ischemic stroke while 13 (7.5%) patients had hemorrhagic stroke. The high rates of strokes in our cohort can be explained by that (1) the main indication for VA-ECMO was postcardiotomy cardiogenic shock (88%) with prolonged cardiopulmonary bypass and aortic clamping times; (2) the studied patients were high risk indicated by baseline SOFA and SAVE scores; (3) the low threshold to do emergency brain CT in our center and we did not include any patient who did not have a brain CT scan.

Our results are like those of Chapman et al. [11] who conducted a study on 412 patients supported by 3 types ECMO had incidence of ischemic stroke 7.0% and ICH 3.4% in all groups, but in the VA-ECMO group, the total CNS complications were 18% which is comparable to our study. Shoskes et al. conducted a large meta-analysis enrolled 8211 adult patients with VA-ECMO and reported neurological insults in 19%, ischemic stroke in 10%, ICH in 6%, and subarachnoid hemorrhage in 11% [9]. Also, Lüsebrink et al. [26] retrospectively analyzed 598 adult patients and reported that 70 (12%) patients developed ICH during VA-ECMO support. On the other hand, Lorusso et al. [8] examined 4522 adult patients with VA-ECMO and reported that 161 (3.6%) patients had ischemic stroke, and 80 patients (1.8%) patients had ICH. Omar et al. [27] studied 171 subjects with ECMO and reported that 10 (5.8%) patients developed ischemic stroke. Le Guennec et al. [4] studied 878 patients and reported that 42 (5.3%) patients had ischemic strokes, and 20 (2.8%) patients had ICH. Almajed et al. studied 244 patients with peripheral VA-ECMO and reported stroke in 14.7% of patients without an increase in hospital mortality [12].

The actual incidence of neurological complications might be underestimated as all studies were retrospective analyses. Many patients could have died with multiorgan system failure without undergoing neuroimaging. Additionally, the absence of regular standardized neurological monitoring may have concealed the true occurrence of these complications [3]. Moreover, postmortem reports showed that up to 90% of VA-ECMO nonsurvivors had cerebral infarcts that could be undiagnosed because of defective monitoring, imaging limitations with clinically silent strokes [15–17].

According to our results, the patients who developed stroke had significantly higher ICU stay, higher rate of CRRT, and higher mortality in comparison to the patients who did not develop stroke. Subgroup analysis revealed a higher mortality in patients with ICH compared to those with ischemic stroke. Cho et al. reported mortality rates of 76% and 86% in patients with ischemic strokes and ICH, respectively, compared to 56% in patients without stroke on VA-ECMO [14]. Shoskes et al. meta-analysis reported 90-day mortality rates of 65% and 72% in patients with ischemic stroke and ICH, respectively, which were significantly higher than those patients without stroke (*p* < 0.0001) [9]. Chapman et al. [11] reported a mortality rate of 56% in patients with stroke compared to 32% in patients without stroke (*p* < 0.001). Also, Lorusso et al. [8] reported mortality rates of 89% and 57% in patients with and without CNS complications, respectively (*p* < 0.001). Le Guennec et al.[4] showed that ICH rather than ischemic stroke was associated with increased mortality (90% vs. 57%, *p* = 0.03). Lüsebrink et al. [26] showed in-hospital mortality rates of 81% and 63% in patients with and without ICH, respectively (*p* = 0.002). A recent small study reported a mortality rate of 90.9% with ICH during ECMO support [28]. On the contrary, a single center study reported that stroke was not associated with a significant increase in mortality or other worse outcomes [12].

Changes in cerebral autoregulation and blood flow, coupled with coagulopathy (arising from shock state or anticoagulation), can increase the likelihood of neurological injury in ECMO patients [29]. It is challenging to discern whether neurological injury is attributable to ECMO support itself or to the primary disease (such as shock state or multiorgan dysfunction) and its management (including vasoactive drugs and mechanical ventilation).

There are many factors before and after VA-ECMO initiation that can cause acute brain injury and still with limited data. These factors include target MAP, blood gases changes, and body temperature. No data exist about optimal MAP to maintain cerebral perfusion, prevent brain insult after ECMO initiation, and target MAP in patients with diagnosed strokes [18]. Moreover, cerebral autoregulation is impaired in patients with nonpulsatile flow which contributes to brain injury [30]. Keeping a higher MAP to maintain cerebral perfusion increases cardiac afterload delaying recovery and may result in ICH especially with anticoagulation [18]. Individualized MAP was recommended according to patients' comorbidities and neurological monitoring with avoidance of hypotension according to the current ELSO consensus guidelines [18]. Hypercapnia is common before ECMO initiation, and results in cerebral vasodilatation impairing cerebral autoregulation and rapid correction after ECMO may lead to cerebral vasoconstriction and brain ischemia [31]. Rapid decline of PaCO2 was linked to risk of stroke, and it was recommended to avoid large decline after ECMO cannulation [4, 18]. Hyperoxia (mild: PaO2 > 100 mmHg; severe: PaO2 > 300 mmHg) after ECMO cannulation was linked to worse neurological outcomes and should be avoided [14, 18, 32]. So, the peri-cannulation time is very crucial that needs early stabilization of hemodynamics, keeping optimal MAP, gradual correction of blood gases with continuous neurological monitoring. Clinical assessment including pupillary reflexes may be inappropriate in the presence of sedation and muscle paralysis. Frequent sedation interruptions and assessment with low threshold to do brain imaging were recommended for early identification of stroke that need timely intervention like anticoagulation holding, mechanical thrombectomy, and neurosurgical procedure [18].

Various studies have identified multiple risk factors for neurological complications in ECMO patients, which may be due to variations in ECMO centers, including the devices used, anticoagulation protocols, the frequency of cranial CT scans, and individual patient differences [3].

In our study, blood lactate at ECMO initiation, LC-T12 h, and CPB time were the predictors of ischemic stroke. Lower BMI and thrombocytopenia were the predictors of ICH. The increase of blood fibrinogen level was protective against ICH but without a statistical significance. Lorusso et al. [8] showed that age, cardiac arrest before ECMO, the use of inotropes, and occurrence of hypoglycemia were associated with neurological complications. Hyperlactatemia and delayed LC were linked to worse neurological outcomes and increased mortality with different cut-off points [10, 25, 33, 34]. Omar et al. [27] described blood lactate ≥ 10 mmol/L before ECMO initiation as a predictor of ischemic stroke after ECMO initiation.

Le Guennec et al. [4] reported central VA-ECMO, female sex, and platelets < 100 (10^9^/L) as predictors of ICH while platelet count > 350 (10^9^/L) as a predictor of ischemic stroke during VA-ECMO use. Our study did not find different rates of strokes according to the cannulation approach of VA-ECMO. Our results are similar to Nishikawa et al. report [35] but different than other reports that revealed increased strokes with central VA-ECMO [4, 10]. Central VA-ECMO may be associated with left ventricular and aortic root thrombosis that can result in cerebral thromboembolism. Also, peripheral VA-ECMO increases cardiac afterload that can result in aortic valve closure and aortic root thrombosis. Left ventricular venting helps to decrease this risk together with systemic anticoagulation [36]. Data on appropriate anticoagulation are deficient especially with the presence of thrombocytopenia and coagulopathy due to ECMO circuit and after cardiac surgery. Moreover, there are not sufficient data on safe anticoagulation reversal in the presence of bleeding and timing to resume it.

Lüsebrink et al. [26] revealed that ICH was positively associated with lactate level (OR: 1.06, 95% CI 1.01–1.11, *p* = 0.02), and diabetes mellitus (OR: 2, 95% CI:1.11–3.56, *p* = 0.02) and negatively associated with fibrinogen (OR: 0.64, 95% CI: 0.49–0.83, *p* < 0.001) and platelet count (OR: 0.32, 95% CI 0.15–0.59, *p* = 0.001). Zhu et al. reported the hypofibrinogenemia and thrombocytopenia in patients with ICH and initiated a predictive model of ICH using platelet count and need for fresh frozen plasma transfusion [28].

Finally, brain injury is very common and multifactorial. There are many challenges in neuromonitoring and care during VA-ECMO with limited evidence for optimal hemodynamics and anticoagulation goals. Clinicians should keep meticulous neuromonitoring and a low threshold for neuroimaging for early detection of strokes. Multidisciplinary teamwork is crucial to delivering individualized care. Many areas are still under research to improve management and overcome challenges like brain imaging modalities. Doing a brain CT scan is sometimes difficult due to risky transfer of critically ill patients, and the sensitivity of CT scan to detect brain ischemia is limited. Recently, low-field portable magnetic resonance imaging was used safely in ICU for ECMO patients [37, 38].

## 5. Conclusions

Ischemic and hemorrhagic strokes are frequent with VA-ECMO support and associated with worse outcomes, especially the hemorrhagic type. Awareness of the incidence and the factors associated with strokes is crucial in early identification and management.

### 5.1. Limitations

Our study is a retrospective single center work. Brain imaging was done for patients who developed neurological manifestations which could result in underreporting subclinical brain insults. We could not add significant laboratory findings like peri-cannulation PaO2 and PaCO2 due to missing data.

## Figures and Tables

**Table 1:** Clinical characteristics of study patients.

Variables		All patients (*n* = 173)	Stroke group (*n* = 35, 20.2%)	No-stroke group (*n* = 138, 79.8%)	*p* value
Age (years)		48 (31, 56)	39 (27, 56)	45 (31,56)	0.25

Sex, male (*n*, %)		98 (56.6)	22 (62.9)	76 (55.1)	0.41

Body mass index (kg/m^2^)		26.5 (22.6, 32.1)	25.5 (20.1, 28.7)	25.9 (22.3, 32)	0.19

Diabetes mellitus (*n*, %)		56 (32.4)	7 (20)	49 (35.5)	0.08

Systemic hypertension (*n*, %)		58 (33.5)	12 (34.3)	46 (33.3)	0.92

Coronary artery disease		22 (12.7)	5 (14.3)	17 (12.3)	0.78

Chronic kidney disease (*n*, %)		35 (20.2)	5 (14.3)	30 (21.7)	0.33

Hemodialysis-dependency (*n*, %)		8 (4.6)	2 (5.7)	6 (4.3)	0.66

SOFA score (SOFA-1)		11.5 (10, 14)	11 (10, 13)	12 (9, 15)	0.28

SAVE score		−1 (−3, 3)	0 (−3, 3)	−1 (−5, 3)	0.45

Old cerebrovascular stroke (*n*, %)		14 (8.1)	4 (11.4)	10 (7.2)	0.49

Previous cardiotomy (*n*, %)		82 (47.4)	16 (45.7)	66 (47.8)	0.82

Atrial fibrillation (*n*, %)		56 (32.4)	11 (31.4)	45 (32.6)	0.89

IABP use (*n*, %)		30 (17.3)	7 (20)	23 (16.7)	0.64

Heart disease (*n*, %)	Rheumatic heart disease	57 (32.9)	8 (22.9)	49 (35.5)	0.56
	Idiopathic cardiomyopathy	46 (26.6)	11 (31.4)	35 (25.4)	
	Ischemic cardiomyopathy	30 (17.3)	6 (17.1)	24 (17.4)	
	ACHD	15 (8.7)	5 (14.3)	10 (7.2)	
	Other	23 (13.3)	5 (14.3)	18 (13)	

Cardiac surgery (*n*, %)		152 (87.9)	30 (85.7)	122 (88.4)	0.11

Cardiac surgery (n, %)	CABG	8 (5.26)	2 (5.71)	6 (4.92)	0.58
	Valve surgery	71 (46.71)	14 (40)	57 (46.7)	
	CABG + valve surgery	18 (11.84)	3 (8.57)	15 (12.3)	
	Heart transplantation	24 (15.78)	4 (11.42)	20 (16.4)	
	Aortic surgery	9 (5.92)	4 (11.42)	5 (4.1)	
	Lung transplantation	15 (9.87)	3 (8.57)	12 (9.84)	
	Pulmonary endarterectomy	1 (0.7)	0	1 (0.08)	
	LVAD insertion	6 (3.9)	0	6 (4.9)	

CPB time (min)		218 (167, 317)	228 (169, 319)	198 (157, 270)	0.203

ACC time(min)		145 (105, 174)	145 (108, 5167.5)	145.5 (104, 5181)	0.87

Cannulation approach (*n*, %)	Central	87 (50.3)	20 (57.1)	67 (48.6)	0.33
	Peripheral	76 (43.93)	12 (34.29)	64 (46.38)	
	Central then peripheral	6 (3.47)	1 (2.86)	5 (3.62)	
	Peripheral then central	4 (2.31)	2 (5.71)	2 (1.45)	

Abbreviations: ACC, aortic cross-clamping; ACHD, adult congenital heart disease; CPB, cardiopulmonary bypass time; ECPR, extracorporeal cardiopulmonary resuscitation; IABP, intra-aortic balloon pump; SAVE, survival after venoarterial ECMO; SOFA, sequential organ failure assessment.

**Table 2:** Laboratory variables and SOFA scores of the patients studied.

Variables	Stroke group	No-stroke group	*p* value
*At ECMO initiation*			
Hemoglobin (gm/L)	95 (84, 108)	100 (88, 112)	0.35
Platelet count (10^9^/L)	103 (53, 157)	97 (59, 182)	0.71
Serum creatinine (μmol/L)	113 (86, 138)	105 (66, 152)	0.48
Serum bilirubin (μmol/L)	25.8 (13.4, 53.9)	35.4 (17.2, 69.7)	0.27
Base excess (mmol/L)	−5.8 (−10.2, −4.1)	−6.95 (−10.5, −4.6)	0.27
Blood HCO3 (mmol/L)	18.9 (17.1, 21.2)	18.6 (14.9, 20.4)	0.22
INR	1.7 (1.3, 2.1)	1.75 (1.5, 2.3)	0.3
Fibrinogen (g/L)	2.93 (2.37, 4.52)	3.16 (2.48, 4.27)	0.8
ALT (units/L)	38.4 (19, 103.4)	41.75 (23.5, 171)	0.32
AST (units/L)	118.7 (48.9, 247)	119.05 (54.7, 289.7)	0.55
Initial blood lactate (mmol/L)	8.9 (5.5, 11.2)	5.7 (4.6, 11.9)	0.02

*After ECMO support*			
Peak blood lactate (mmol/L)	16.35 (10.9, 20)	17.8 (10.9, 25)	0.04
Blood lactate at T12 (mmol/L)	8.7 (4.7, 14.5)	5.8 (4.6, 15)	0.024
Blood lactate at T24 (mmol/L)	4.1 (2.3, 7)	2.9 (2.4, 11.4)	0.06
Lactate clearance % (LC-T12)	6.35 (−51.5, 40.6)	14.65 (−43.55, 38.3)	< 0.001
Lactate clearance % (LC-T24)	52.08 (5.88, 64.43)	32.59 (−17.76, 65.52)	0.003
Platelet count 48 h (10^9^/L)	81 (59, 124)	88 (72, 138.5)	0.39
Fibrinogen 48 h (g/L)	2.42 (1.91, 3.62)	2.56 (2.24, 3.83)	0.18
The 3^rd^ day SOFA	14 (11, 16)	14 (9, 17)	0.9
The 5^th^ day SOFA	15 (10, 18)	14 (8, 18)	0.74

Abbreviations: ALT: alanine transaminase, AST: aspartate aminotransferase, INR: international normalized ratio; SOFA, sequential organ failure assessment.

**Table 3:** Clinical and laboratory characteristics of the ischemic and hemorrhagic strokes.

Variables	Ischemic stroke group (*n* = 22, 12.7%)	Nonischemic stroke group (*n* = 151, 87.3%)	*p* value	Hemorrhagic stroke group (*n* = 13, 7.5%)	Nonhemorrhagic stroke group (*n* = 160, 92.5%)	*p* value
Age (years)	43 (29, 57)	45 (29, 56)	0.91	29 (25, 51)	45 (31, 56)	0.04
Sex, female (*n*, %)	9 (40.9)	66 (43.7)	0.8	5 (38.5)	70 (43.8)	0.7
BMI (kg/m^2^)	25.9 (22.6, 28.2)	25.7 (21.8, 32)	0.48	22.7 (19.7, 29.2)	25.9 (22.6, 31.6)	0.02
DM (*n*, %)	4 (18.2)	52 (34.4)	0.13	2 (15.4)	54 (33.8)	0.23
Systemic HTN (*n*, %)	7 (31.8)	51 (33.8)	0.86	4 (30.8)	54 (33.8)	1
CKD (*n*, %)	1 (4.5)	34 (22.5)	0.051	3 (23.1)	32 (20)	0.73
Previous stroke (*n*, %)	1 (4.5)	13 (8.6)	1	2 (15.4)	12 (7.5)	0.28
Atrial fibrillation (*n*, %)	6 (27.3)	50 (33.1)	0.58	4 (30.8)	52 (32.5)	1
IABP use (*n*, %)	5 (22.7)	25 (16.6)	0.54	2 (15.4)	28 (17.5)	1
Cardiac surgery (*n*, %)	19 (86.4)	133 (88.1)	0.21	9 (69.2)	143 (89.4.4)	0.24
CPB time (min)	228.5 (169, 319)	179 (148, 241)	0.042	268 (218, 297)	217 (167, 317)	0.39
ACC (min)	147.5 (105, 179)	131.5 (108, 161)	0.36	166 (163, 17 3)	140 (105, 174)	0.28
SAVE score	1 (−4, 3)	−1 (−5, 3)	0.45	−1 (−3, 1)	−0.5 (−5, 3)	0.96
SOFA (D1)	11 (10, 13)	12 (9, 14)	0.31	12 (11, 13)	12 (9, 14)	0.81
SOFA (D3)	13 (11, 15)	14 (10, 17)	0.39	15 (14, 16)	14 (10, 17)	0.43
SOFA (D5)	13 (9, 16)	15 (8, 18)	0.54	16 (13, 18)	15 (8, 18)	0.35
Initial platelet count (10^9^/L)	106 (60, 188)	98 (56, 176)	0.5	98 (53, 115)	98.5 (59, 182.5)	0.18
Platelet count- 48 h (10ˆ^9^/L)	89 (69, 136)	91 (72, 141.2)	0.46	32 (28, 67)	78 (54, 121.6)	0.001
Initial fibrinogen (g/L)	3.2 (2.8, 4.2)	3.41 (2.68, 4.6)	0.57	2.82 (2.61, 3.92)	3.1 (2.92, 4.12)	0.61
Fibrinogen 48 h (g/L)	2.81 (2.13, 3.7)	2.36 (2.18, 3.93)	0.42	1.21 (0.8, 1.92)	2.34 (1.74, 2.46)	0.04
Initial blood lactate (mmol/L)	9.1 (5.7, 12)	7.45 (5.1, 9.9)	0.023	9.4 (6.3, 11.5)	8.1 (5.6, 11.8)	0.19
Peak lactate (mmol/L)	18.5 (12.9, 25)	16.4 (10.9, 25)	0.71	16.7 (13.6, 18.6)	16.45 (11.05, 25)	0.73
Lactate at T12 (mmol/L)	8.5 (4.8, 14.8)	5.3 (4.3, 15)	0.04	6.8 (5.3, 9.5)	8.65 (4.65, 15)	0.35
Lactate at T24 (mmol/L)	4.3 (2.3, 10)	2.85 (2.1, 7.2)	0.07	4.1 (2.7, 4.7)	4.4 (2.2, 9.9)	0.72
LC-T12 (%)	−6.81 (−65.29, 41.94)	11.24 (−42.05, 38.3)	< 0.001	1.44 (−50.75, 38.53)	17.19 (−19.97, 40)	0.09
LC-T24 (%)	51.04 (5.88, 67.19)	34.9 (−15.25, 66.67)	0.004	45.48 (−17.37, 67.1)	57.73 (7.48, 64.35)	0.51

Abbreviations: ACC, aortic cross-clamping; CKD, chronic kidney disease; CPB, cardiopulmonary bypass time; DM, diabetes mellitus; IABP, intra-aortic balloon pump; LC, lactate clearance; SAVE, survival after venoarterial ECMO; SOFA, sequential organ failure assessment.

**Table 4:** Clinical outcomes of the patients studied.

Variables	All patients (*n* = 173)	Stroke group (*n* = 35, 20.2%)	Non-stroke group (*n* = 138, 79.8%)	*p* value
ECMO days	7 (4.13)	8 (4.15)	6.5 (3.12)	0.16
ICU days	17 (8.29)	21 (12.38)	15.5 (8.26)	0.03
New need for CRRT (*n*, %)	86 (49.7)	22 (62.9)	64 (46.4)	0.026
On-ECMO mortality (*n*, %)	81 (46.8)	19 (54.3)	62 (44.9)	0.041
Hospital mortality (*n*, %)	115 (66.5)	25 (71.4)	90 (65.2)	0.08
Limb ischemia (*n*, %)	21 (12.2)	5 (14.3)	16 (11.6)	0.77
Bowel ischemia (*n*, %)	7 (4.05)	1 (2.9)	6 (4.3)	1
Bowel surgery (*n*, %)	2 (1.2)	0	2 (1.4)	1
Post-ECMO durable LVAD (*n*, %)	4 (2.3)	0	4 (2.9)	0.58
Post-ECMO heart transplantation (*n*, %)	4 (2.3)	0	4 (2.9)	0.58

Abbreviations: CRRT, continuous renal replacement therapy; ECMO, extracorporeal membrane oxygenation; ICU, intensive care unit; LVAD, left ventricular assist device.

**Table 5:** Clinical outcomes of the ischemic and hemorrhagic strokes in comparison to the whole population.

Variables	Ischemic stroke group (*n* = 22, 12.7%)	Nonischemic stroke group (*n* = 151, 87.3%)	*p* value	Hemorrhagic stroke group (*n* = 13, 7.5%)	Nonhemorrhagic stroke group (*n* = 160, 92.5%)	*p* value
ECMO days	9 (5.5, 15)	6.5 (3, 12)	0.26	10 (6, 11)	7 (3, 12.5)	0.29
ICU days	22.5 (13.5, 38)	16 (8, 31.5)	0.04	21 (11, 38)	17 (8, 32.5)	0.34
New CRRT (*n*, %)	15 (68.2)	71 (47)	0.004	7 (53.8)	79 (49.4)	0.59
On-ECMO mortality (*n*, %)	10 (45.5)	71 (47)	0.29	9 (69.2)	72 (45)	0.03
Hospital mortality (*n*, %)	14 (63.6)	101 (66.9)	0.38	11 (84.6)	104 (65)	0.021

Abbreviations: CRRT, continuous renal replacement therapy; ECMO, extracorporeal membrane oxygenation; ICU, intensive care unit.

**Table 6:** Comparison of clinical outcomes between the ischemic and hemorrhagic strokes.

Variables	Ischemic stroke group (*n* = 22, 12.7%)	Hemorrhagic stroke group (*n* = 13, 7.5%)	*p* value
ECMO days	9 (5.5, 15)	10 (6, 11)	0.5
ICU days	22.5 (13.5, 38)	21 (11, 38)	0.3
New CRRT (*n*, %)	15 (68.2)	7 (53.8)	0.08
On-ECMO mortality (*n*, %)	10 (45.5)	9 (69.2)	0.03
Hospital mortality (*n*, %)	14 (63.6)	11 (84.6)	0.01

Abbreviations: CRRT, continuous renal replacement therapy; ECMO, extracorporeal membrane oxygenation; ICU, intensive care unit.

**Table 7:** Logistic multivariate regression for predicting strokes.

Variables	OR	95% CI	*p* value
*Ischemic stroke*			
Initial blood lactate	1.6	1.2–8.92	0.031
Cardiopulmonary bypass time	1.8	1.32–6.42	0.02
Lactate clearance at 12 h	2.4	1.91–17.4	0.042
Hemodialysis	1.31	1.11–13.6	0.09

*Hemorrhagic stroke*			
BMI	0.21	0.131–0.46	0.02
Platelet count	0.322	0.182–0.783	0.001
Age	0.92	0.69–1.12	0.08
Fibrinogen	0.74	0.48–0.86	0.06

## Data Availability

The data that support the findings of this study are available from the corresponding author upon reasonable request.

## Nomenclature

ECMO: Extracorporeal membrane oxygenation
CRRT: Continuous renal replacement therapy
CPB: Cardiopulmonary bypass
ECPR: Extracorporeal cardiopulmonary resuscitation
LC: Lactate clearance
BMI: Body mass index
ICH: Intracranial hemorrhage
ICU: Intensive care unit
